# Paediatric trigger thumbs: patient-reported outcome measures over a minimum of ten years’ follow-up

**DOI:** 10.1302/2633-1462.59.BJO-2024-0056.R1

**Published:** 2024-09-04

**Authors:** Sebastian Farr, Teofil Mataric, Bettina Kroyer, Sitanshu Barik

**Affiliations:** 1 Department of Pediatric Orthopaedics and Foot and Ankle Surgery, Orthopaedic Hospital Speising, Vienna, Austria; 2 Department of Surgery, Krankenhaus Göttlicher Heiland, Vienna, Austria; 3 Center for Medical Statistics, Informatics and Intelligent Systems, Medical University of Vienna, Vienna, Austria; 4 Department of Orthopedics, All India Institute of Medical Sciences, Deoghar, India

**Keywords:** Thumb, Trigger thumb, Interphalangeal joint, Metacarpophalangeal joint, Pulley release, Trigger fingers, Patient-reported outcome measures (PROMs), QuickDASH, Revision surgery, Disabilities of the Arm, Shoulder and Hand questionnaire, Metacarpophalangeal joint, Interphalangeal joint, Fischer's exact test, Kruskal-Wallis tests, Functional outcomes

## Abstract

**Aims:**

The paediatric trigger thumb is a distinct clinical entity with unique anatomical abnormalities. The aim of this study was to present the long-term outcomes of A1 pulley release in idiopathic paediatric trigger thumbs based on established patient-reported outcome measures.

**Methods:**

This study was a cross-sectional, questionnaire-based study conducted at a tertiary care orthopaedic centre. All cases of idiopathic paediatric trigger thumbs which underwent A1 pulley release between 2004 and 2011 and had a minimum follow-up period of ten years were included in the study. The abbreviated version of the Disabilities of Arm, Shoulder and Hand questionnaire (QuickDASH) was administered as an online survey, and ipsi- and contralateral thumb motion was assessed.

**Results:**

A total of 67 patients completed the survey, of whom 63 (94%) had full interphalangeal joint extension or hyperextension. Severe metacarpophalangeal joint hyperextension (> 40°) was documented in 15 cases (22%). The median QuickDASH score was 0 (0 to 61), indicating excellent function at a median follow-up of 15 years (10 to 19). Overall satisfaction was high, with 56 patients (84%) reporting the maximal satisfaction score of 5. Among 37 patients who underwent surgery at age ≤ two years, 34 (92%) reported the largest satisfaction, whereas this was the case for 22 of 30 patients (73%) with surgery at aged > two years (p = 0.053). Notta’s nodule resolved in 49 patients (73%) at final follow-up. No residual triggering or revision surgery was observed.

**Conclusion:**

Surgical release of A1 pulley in paediatric trigger thumb is an acceptable procedure with excellent functional long-term outcomes. There was a trend towards higher satisfaction with earlier surgery among the patients.

Cite this article: *Bone Jt Open* 2024;5(9):736–741.

## Introduction

The paediatric trigger thumb is a distinct clinical entity with unique anatomical abnormalities.^1^ It can be idiopathic or secondary to central nervous system, storage, and inflammatory disorders. The management varies from initial conservative management by observation or splinting alone to operative management, which includes release of the A1 pulley. Additionally, unlike in adult cases, the willingness to further explore and examine the oblique pulley bundle is needed to avoid partial relief of symptoms or recurrence. This is because of the specific anatomical abnormalities which are more prevalent in the paediatric age group.^2,3^

Previous reports have provided evidence on how to approach paediatric trigger thumbs.^4,5^ Surgery has been shown to have the highest rate of treatment success (95%), followed by splinting (67%) and exercising alone (55%).^4^ However, the literature lacks long-term studies assessing objective outcomes on the basis of dedicated patient-reported outcome measures (PROMs). The aim of this study was therefore to present the outcomes of A1 pulley release in idiopathic paediatric trigger thumbs based on established PROMs.

## Methods

This study was a cross-sectional questionnaire-based study conducted at a tertiary care orthopaedic institution (Orthopaedic Hospital Speising, Austria). Patients were referred to the centre by general orthopaedists and/or paediatricians across the country to assess the child’s thumb pathology. Surgery (exclusively open A1 pulley release) was recommended for cases showing a fixed thumb interphalangeal (IP) joint flexion contracture or those non-responsive to conservative treatment. All cases of idiopathic paediatric trigger thumbs (aged < 18 years) who underwent A1 pulley release from 2004 to 2011 and had a minimum follow-up period of ten years were included in the study. Secondary paediatric trigger thumbs arising due to central nervous system or storage disorders or inflammatory conditions, and trigger fingers were excluded from the study. Institutional ethical clearance was obtained for the study (EK13-2022).

### Data collection

The identification of the study subjects was conducted through an electronic patient database. The patients were then contacted and invited through mail, both postal and electronic, explaining the study purpose. PROM forms and a clinical questionnaire about the current ipsi- and contralateral thumb motion were distributed and filled out by the patients themselves in an online sheet. Thumb motion was stratified in the instructions of the online sheet into magnitude of IP joint extension, metacarpophalangeal (MCP) joint extension, and comparison to contralateral MP (cMP) joint extension, and if applicable hypertension thereof. For IP extension, survey respondents were asked to grade extension as: 1) lack of extension of 40° and more; 2) lack of 20°; 3) full extension to 0°; or 4) > 0°/hyperextension. The data and consent of the willing subjects were recorded. The demographic data such as age at surgery, duration of symptoms, and any potential complications, as well as current clinical situation, were recorded (see Supplementary Material). Patient satisfaction was recorded on a Likert scale of 1 to 5 points, with 5 being the maximum satisfaction rate.

### PROMs

The abbreviated version of the Disabilities of the Arm, Shoulder and Hand (QuickDASH)^6^ questionnaire was used in the study. It consists of 11 items which cover the difficulties in various daily activities, including pain, activity-associated pain, tingling, weakness, and stiffness, along with the impact of the disease on social life, work, sleep, and satisfaction.^6,7^ The score varies from 0 with no disability to 100 with maximum disability.

### Statistical analysis

For descriptive purposes, median, minimum, and maximum were calculated for metric variables, and absolute and relative frequencies were calculated for categorical variables. Kruskal-Wallis tests were applied to assess differences in the QuickDASH score with regard to different levels of extension, pain, MP extension, cMP extension, MP pain, and satisfaction. Boxplots were created to visualize the distribution of QuickDASH by stages of other variables. For the satisfaction score, the largest and most frequent value of 5 was used to define two groups as satisfaction < 5 and satisfaction = 5. Proportions of patients with satisfaction = 5 were compared between subgroups using Fisher’s exact test. Bar plots were created to visualize the relative frequencies. Two groups with age at surgery ≤ two years and > two years were defined using the median age at surgery of two years as cut-off. Further analyses were conducted as described for satisfaction. A p-value of 0.05 was considered to indicate statistical significance. Due to the explorative character of the study, no adjustment for multiple testing was applied. All statistical analyses were performed using R v. 4.0.2 (R Foundation for Statistical Computing, Austria).

## Results

Among the total cohort of 131 cases fulfilling the eligibility criteria, 67 patients responded to the follow-up invitation (51%). The median age at surgery was two years (0 to 16) and mean age at follow-up was 15 years (10 to 19).

Full IP extension (or hyperextension) was observed in 63 patients (94%). Severe MP hyperextension (> 40°) was documented in 15 cases (22%). Overall, 33 patients (62%) stated that their operated thumb MP extension was the same as the contralateral, healthy thumb, while 20 patients (38%) stated that it was more extendable. No residual triggering or revision surgery was observed. All patient characteristics and clinical results are summarized in Table I and Table II.

**Table I.** Demographic patient characteristics.*

**Variable**	**Data**
**Sex, n (%)**	
Female	35 (52)
Male	32 (48)
Median age at surgery, yrs (range)	2 (0 to 16)
Mean follow-up time, yrs (range)	15 (10 to 19)
**Side affected, n (%)**	
Both	11 (16)
Left	34 (51)
Right	22 (33)
Initial extension deficit, ° (n = 33)	20 (5 to 90)

* All n = 67, unless otherwise stated.

**Table II.** Clinical patient characteristics and outcomes.*

Variable	Data
Satisfaction, n (%)	
2	1 (1)
3	4 (6)
4	6 (9)
5	56 (84)
**Difference to contralateral side?, n (%)**	
No	56 (84)
Yes	11 (16)
Median pain (range)	0 (0 to 6)
**Function, n (%)**	
IP extension -40° and more	3 (4)
IP extension -20°	1 (1)
IP extension 0°	23 (34)
IP extension > 0°/hyperextension	40 (60)
MP extension 0°	19 (28)
MP extension 10 to 20°	14 (21)
MP extension 20 to 40°	19 (28)
MP extension > 40°	15 (22)
cMP extension 0 (same as contralateral thumb)	33 (62)
cMP extension 1	2 (4)
cMP extension 2	9 (17)
cMP extension 3	5 (9)
cMP extension 4	3 (6)
cMP extension 5 (much more than contralateral thumb; n = 53)	1 (2)
MP pain 0	40 (62)
MP pain 1	11 (17)
MP pain 2	9 (14)
MP pain 3 (n = 64)	4 (6)
**Notta nodule present?, n (%)**	
No	49 (73)
Yes	18 (27)
**Outcome score**	
Median DASH score (range)	0 (0 to 61.36)
**Work restricted?, n (%)**	
Don't know	3 (5)
No	61 (92)
*Yes* (n = 66)	2 (3)
**Sports restricted?, n (%)**	
Don't know	3 (4)
No	64 (96)

* All n = 67, unless otherwise stated.

cMP, contralateral metacarpophalangeal; DASH, Disabilities of the Arm, Shoulder and Hand questionnaire; IP, interphalangeal; MP, metacarpophalangeal.

### QuickDASH score

Across all 67 included patients, the median QuickDASH score was 0 (0 to 61) (Table III). QuickDASH scores were significantly lower for patients without pain (median 0; 0 to 61) compared to patients with a pain score > 0 (median 6; 2 to 34; p < 0.001, Kruskal-Wallis test). Moreover, patients with satisfaction ≥ 5 had significantly lower QuickDASH scores (median 0; 0 to 61) than patients with satisfaction < 5 (median 5; 0 to 34; p = 0.004, Fisher’s exact test). No significant differences in QuickDASH were found between different extension levels (p = 0.080, Kruskal-Wallis test); however, the small number of subjects with an extension lack of 20° to 40° limits the interpretation of this comparison. QuickDASH scores appeared similar across levels of MP extension and cMP extension and no significant differences were found. Observed QuickDASH scores were increasing with increasing level of MP pain; however, these differences were not statistically significant (p = 0.075, Kruskal-Wallis test).

**Table III.** Association of DASH score with IP extension, pain, MP and cMP extension, MP pain, and satisfaction.

Variable	N	Median (range)	p-value*
IP extension -40° and more	3	5 (5 to 5)	
IP extension -20°	1	0 (0 to 0)	
IP extension 0°	23	0 (0 to 30)	
IP extension > 0°/hyperextension	40	0 (0 to 61)	0.080
Pain 0	61	0 (0 to 61)	
Pain > 0	6	6 (2 to 34)	**< 0.001**
MP extension 0°	19	0 (0 to 30)	
MP extension 10 to 20°	14	0 (0 to 23)	
MP extension 20 to 40°	19	0 (0 to 61)	
MP extension > 40°	15	0 (0 to 7)	0.305
cMP extension 0	33	0 (0 to 61)	
cMP extension 1	2	13 (2 to 23)	
cMP extension 2	9	0 (0 to 30)	
cMP extension 3	5	0 (0 to 34)	
cMP extension 4	3	2 (0 to 9)	
cMP extension 5	1	0 (0 to 0)	0.369
MP pain 0	40	0 (0 to 61)	
MP pain 1	11	2 (0 to 9)	
MP pain 2	9	5 (0 to 30)	
MP pain 3	4	11 (0 to 34)	0.075
Satisfaction < 5	11	5 (0 to 34)	
Satisfaction 5	56	0 (0 to 61)	**0.004**

Significant p-values are in bold.

* Kruskal-Wallis test.

cMP, contralateral metacarpophalangeal; DASH, Disabilities of the Arm, Shoulder and Hand questionnaire; IP, interphalangeal; MP, metacarpophalangeal.

### Patient satisfaction

**Fig. 1:**
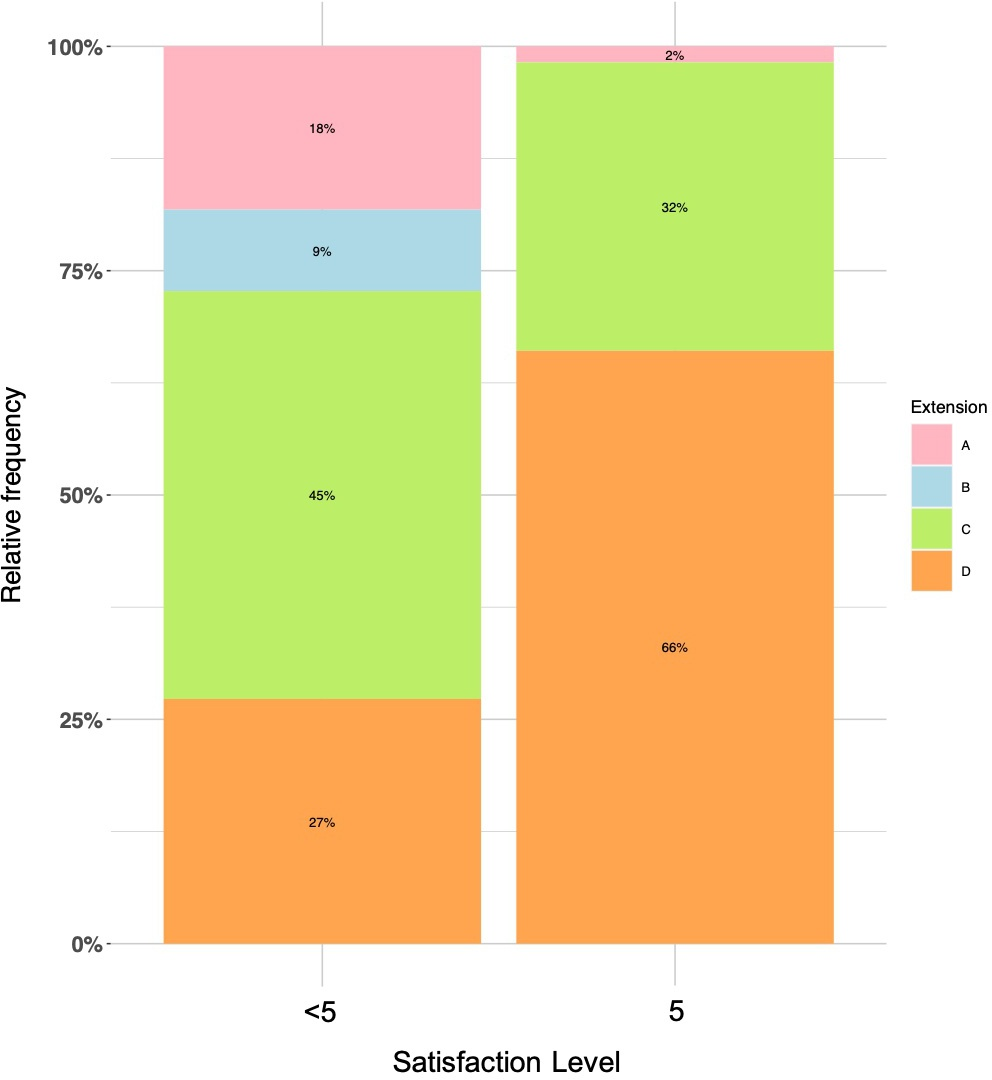
Barplot is showing differences of satisfaction level (5 vs < 5) depending on the magnitude of interphalangeal joint extension. a) Lack of extension of 40° and more; b) lack of 20°; c) full extension to 0°; d) > 0°/hyperextension.

Overall satisfaction was high, with 56 patients (84%) reporting the maximum satisfaction score of 5. Lower scores of 4, 3, and 2 were reported by six (9%), four (6%), and one (1%) patient, respectively. No patient had a satisfaction score of 1 (Table II). Satisfaction was significantly associated with a higher degree of IP extension (p = 0.008; Figure 1), less pain (p < 0.001), and less MP pain (p = 0.003, all Fisher's exact test). In particular, 55 out of 61 patients who were free of pain had the largest satisfaction score of 5. In contrast, only one out of six patients with pain also reported a satisfaction of 5. There was no significant association between satisfaction and MP hyperextension (p = 0.675, Fisher's exact test)) or cMP hyperextension (p = 0.158, Fisher's exact test); Table IV).

**Table IV.** Association of satisfaction with IP extension, pain, MP and cMP extension, and MP pain.

Variable	N	Satisfaction 5, n (%)	Satisfaction < 5, n (%)	p-value*
IP extension -40° and more	3	1 (2)	2 (18)	
IP extension -20°	1	0 (0)	1 (9)	
IP extension 0°	23	18 (32)	5 (45)	
IP extension > 0°/hyperextension	40	37 (66)	3 (27)	**0.008**
Pain 0	61	55 (98)	6 (55)	
Pain > 0	6	1 (2)	**5 (45)**	**< 0.001**
MP extension 0°	19	16 (29)	3 (27)	
MP extension 10 to 20°	14	11 (20)	3 (27)	
MP extension 20 to 40°	19	15 (27)	4 (36)	
MP extension > 40°	15	14 (25)	1 (9)	0.675
cMP extension 0	33	30 (68)	3 (33)	
cMP extension 1	2	1 (2)	1 (11)	
cMP extension 2	9	6 (14)	3 (33)	
cMP extension 3	5	4 (9)	1 (11)	
cMP extension 4	3	2 (5)	1 (11)	
cMP extension 5	1	1 (2)	0 (0)	0.158
MP pain 0	40	38 (72)	2 (18)	
MP pain 1	11	7 (13)	4 (36)	
MP pain 2	9	5 (9)	4 (36)	
MP pain 3	4	3 (6)	1 (9)	**0.003**

Significant p-values are in bold.

* Fisher’s exact test.

cMP, contralateral metacarpophalangeal; IP, interphalangeal; MP, metacarpophalangeal.

### Age at surgery

Median age at surgery was two years (0 to 16). In total, 37 patients (55%) were aged ≤ two years at surgery and 30 (45%) were aged > two years at surgery. There was a trend of better satisfaction with surgery at lower age, but this was not statistically significant. Among 37 patients who underwent surgery at aged ≤ two years, 34 (92%) reported the largest satisfaction, whereas this was the case for 22 out of 30 patients (73%) with surgery aged > two years (p = 0.053). The variables IP extension, MP extension, and MP pain each showed similar patterns among patients with age at surgery ≤ two years versus > two years, and any between-group differences were not significant (Table V).

**Table V.** Association of age at surgery with satisfaction, IP extension, MP extension, and MP pain.

Variable	N	Aged ≤ two years, n (%)	Aged > two years, n (%)	p-value*
Satisfaction < 5	11	3 (8)	8 (27)	
Satisfaction 5	56	34 (92)	22 (73)	0.053
IP extension -40° and more	3	1 (3)	2 (7)	
IP extension -20°	1	1 (3)	0 (0)	
IP extension 0°	23	11 (30)	12 (40)	
IP extension > 0°/hyperextension	40	24 (65)	16 (53)	0.566
MP extension 0°	19	9 (24)	10 (33)	
MP extension 10 to 20°	14	7 (19)	7 (23)	
MP extension 20 to 40°	19	12 (32)	7 (23)	
MP extension > 40°	15	9 (24)	6 (20)	0.737
MP pain 0	40	23 (68)	17 (57)	
MP pain 1	11	6 (18)	5 (17)	
MP pain 2	9	4 (12)	5 (17)	
MP pain 3	4	1 (3)	3 (10)	0.652

* Fisher’s exact test.

cMP, contralateral metacarpophalangeal; IP, interphalangeal; MP, metacarpophalangeal.

## Discussion

This study aimed to analyze the long-term quality of life after surgery for paediatric trigger thumb. The results confirm that surgical intervention obtains excellent long-term functional outcomes well into adulthood.

The median QuickDASH score noted in this study was 0, and 94% reported full IP motion of their affected thumb. A total of 61 patients (91%) had no pain and 56 patients (84%) had a satisfaction score of 5. Moreover, better IP extension was associated with better satisfaction but not improved functional outcomes in QuickDASH score compared to cases with less-than-full IP extension. Causes for less-than-full IP extension could theoretically be either incomplete A1 division or longstanding trigger thumb deformity, leading to eventual capsular contracture.

Interestingly, 38% of patients still reported an increased amount of MP hyperextension compared to the healthy side, likely caused by compensatory mechanisms in childhood prior to surgical intervention. However, no correlation between IP and MP extension was found. Overall, 50% of patients had more than 20° and 22% more than 40° of MP hyperextension, respectively. Intermittent pain in the MP joint, present in as much as 38%, also affected the eventual satisfaction rate negatively. Unfortunately, we did not find comparable literature with regards to the degree of residual motion after surgery and functional outcomes.

The earliest report of surgical outcomes in paediatric trigger thumb dates back to 1974: the study, by Dinham and Meggitt,^8^ included 131 thumbs in 105 patients. Overall, 80% of patients (105/131) needed surgical intervention in the form of an A1 pulley release, and of these, 95.2% (100/105) had full recovery of the motion at the IP joint. The study also noted a spontaneous resolution rate of 12% until six months after birth. Since that study, more and more surgeons have been opting for surgical intervention in fixed trigger thumbs given that it is a minor procedure with very low complication rates.^9^ The numbers vary from a surgery recommendation rate of 62% to 66% for an 18-month-old child to 81% to 90% for a three-year-old child.^9^ Our results show that these excellent outcomes are sustainable over time into adulthood.

Marek et al^10^ included 217 thumbs in 173 patients in a retrospective review of the outcomes of surgical release in paediatric trigger thumb. At 27-day follow-up, there was an improvement of extension at the IP joint by a mean of 35°. At a follow-up period of 4.2 years, all the parents were satisfied with the outcomes of the surgical intervention. The study also tried to survey the paediatric hand surgeons regarding their practice patterns. The pattern indicated that 85% of the interviewed surgeons would surgically release the A1 pulley in a two year old if the IP joint was locked, whereas 52% of would manage it conservatively if the triggering was intermittent. The opinion was divided in the case of intermittent triggering, whereas it was more unified in the case of locked trigger thumb. A more recent study by Hutchinson et al^11^ found that those with an initial IP joint flexion of 30° or less had increased chances of spontaneous resolution at three years, but those with more than 30° should be advised for early surgical release.

In our study, 92% of patients operated on under the age of two years had better satisfaction rates but similar function, compared to 73% of patients above the age of two years. Although this was only borderline statistically significant, it is an interesting finding and may be related to other influencing factors. In the existing literature, surgery has been shown to be reliable even in those older than three years of age.^12^ However, no such comparable study exists evaluating the surgical age cut-off and PROMs. Most studies based their age recommendations purely on whether or not full IP extension and/or complications were observed.

The long mean follow-up of 15 years with a sample size of 67 in this study validates the results of surgical intervention in paediatric trigger thumb and is, to the best of our knowledge, the longest reported so far, along with the one published by McAdams et al.^13^ While these authors had a follow-up ranging from two to 40 years, our study provided long-term outcomes with a minimum follow-up of ten years. Additionally, our study is one of the first in the literature to have PROMs in the form of QuickDASH scores to report the outcomes of A1 pulley release. The data collection for this study involved contacting the patients both by electronic as well as physical mail; the use of both methods helped to minimize the number of patients who may have missed the study. Despite its strengths, the study is limited by its retrospective nature, which is characterized by selection bias. The bias of the surgeon, as well as of the parents in opting for the surgical treatment, cannot be ruled out. Future studies should be done to establish the ideal age for surgery.

Surgical release of the A1 pulley in paediatric trigger thumb is a durable procedure with excellent functional long-term outcomes and negligible complication rates if done in a specialized centre. The benefits of the surgery are maintained into adolescence and adulthood. There was a trend towards higher satisfaction with earlier surgery among the patients.

**Take home message**

- Long-term results after surgical trigger thumb release provide excellent and long-lasting results with regard to function and patient satisfaction.

- Surgery performed under the age of two years had a tendency to be beneficial with regard to overall patient satisfaction rate.

## Data Availability

All data generated or analyzed during this study are included in the published article and/or in the supplementary material.
